# Draft genome of *Streptococcus agalactiae* serotype Ia FBC260 causing hemorrhagic septicemia with massive cellular meningitis in cultured Nile tilapia from the Philippines

**DOI:** 10.1128/mra.00911-24

**Published:** 2024-12-09

**Authors:** Casiano H. Choresca, Francis S. Legario, Ma. Elaine E. De Leon, Mary Nia M. Santos, Benjamin E. Bumanlag, Christine Grace P. Ca-as, Angelie A. Gente, Paul Christian T. Gloria

**Affiliations:** 1 Department of Agriculture, National Fisheries Research and Development Institute-Fisheries Biotechnology Center, Science City of Muñoz, Nueva Ecija, Philippines; 2 Natural Sciences Department, College of Arts and Sciences, Iloilo Science and Technology University, Iloilo City, Iloilo, Philippines; 3 Natural Sciences Research Institute, University of the Philippines, Quezon City, Metro Manila, Philippines; University of Maryland School of Medicine, Baltimore, Maryland, USA

**Keywords:** *Streptococcus agalactiae*, tilapia, aquaculture

## Abstract

We report the draft genome of *Streptococcus agalactiae* serotype Ia strain FBC260, associated with hemorrhagic septicemia and massive cellular meningitis in cultured Nile tilapia from the Philippines. This genomic resource of *S. agalactiae* from the Philippines will provide valuable insights for disease management.

## ANNOUNCEMENT

*Streptococcus agalactiae*, commonly referred to as Group B *Streptococcus*, is a significant bacterial pathogen associated with hemorrhagic septicemia with subsequent meningitis or meningoencephalitis in tilapia worldwide (1–3). Streptococcosis poses serious threats to the aquaculture industry due to the high mortality rate causing heavy economic losses (4). In the Philippines, two serotypes are circulating among tilapia farms, namely, *S. agalactiae* Ia (hemolytic and more prevalent) and *S. agalactiae* Ib (non-hemolytic and less prevalent) (1). Notably, various species of both marine and freshwater fish, such as golden pompano (*Trachinotus blochii*), rainbow trout (*Oncorhynchus mykiss*), and channel catfish (*Ictalurus punctatus*), have been reported as susceptible hosts to *S. agalactiae* infection (5–7). Despite numerous studies exploring the genome of this pathogen across various hosts, research focusing on isolates from Nile tilapia in the Philippines remains limited. The *S. agalactiae* serotype Ia FBC260 was isolated from the spleen of moribund tilapia collected in Talisay, Batangas, Philippines. The bacterial isolation was done at the Institute of Aquaculture, University of Stirling, United Kingdom, and has been approved by the Animal Welfare and Ethical Review Body of University of Stirling (AWERB: (18/19) 141).

Pure culture of the isolate was cultured on brain heart infusion agar and incubated at 28°C for 48 hours for both maintenance and DNA extraction. Bacterial colonies were scraped and subjected to genomic DNA extraction using Maxwell Prokaryote/Eukaryote SEV DNA purification kit and Maxwell 16 MDx instrument. The genome sequencing library was prepared using the TruSeq Nano DNA kit and sequenced on an Illumina HiSeq X Ten platform with 151 bp, paired-end settings. All bioinformatics tools used were run in default unless otherwise indicated. Raw reads were adapter-trimmed and quality-filtered to Phred20 using Trimmomatic v0.36 (8). Assembly was carried out using Platanus-allee v2.2.2 (9) using 21-mers as the optimal parameter.

Sequencing produced 9,200,420 raw reads, and assembly yielded a genome size of 2,103,013 bp, 35.44% GC, N_50_ of 77,187, and sequencing depth of 565×, comprising 46 contigs. Taxonomic identification using average nucleotide identity, through pyANI v.0.2 (10) showed 99.99% similarity to *S. agalactiae* CS2108 (GCF_029917085.1)*,* a reference genome, confirming the species identity. BUSCO (11) indicated 98.8% completeness of the draft genome – showing near-complete capture of conserved orthologs. For annotation, Prodigal (12), RNAmmer (13), Aragorn (14), SignalP (15), and Infernal (16) were used. Annotation identified 2,086 genes, including 2,043 protein-coding sequences, 40 tRNA, 2 rRNA, and 1 tmRNA genes. Further functional annotation using EggNOG (17) showed a high number of genes related to carbohydrate metabolism and protein biogenesis. However, the most abundant pathway is linked to those with unknown functions, suggesting the need for further studies to characterize gene function in this genome.

## Data Availability

This genome project has been registered at NCBI under accession number PRJNA1122308. Raw reads were deposited at the NCBI Sequence Read Archive (SRA) under the accession number SRR29353182. The genome sequence has been deposited at GenBank under the accession number JBEGDS000000000. The annotation file can be accessed through Zenodo (https://doi.org/10.5281/zenodo.13766566).
